# Impact of Bariatric Surgery and Endoscopic Therapies on Liver Health in Metabolic Dysfunction-Associated Steatotic Liver Disease: A Review

**DOI:** 10.3390/jcm14124012

**Published:** 2025-06-06

**Authors:** Dana Tasabehji, Sanaz Saleh, Mohamad Mokadem

**Affiliations:** 1 Department of Internal Medicine, Roy J. and Lucille A. Carver College of Medicine, University of Iowa, Iowa City, IA 52242, USA; 2 Fraternal Order of Eagles Diabetes Research Center, University of Iowa, Iowa City, IA 52242, USA; 3 Obesity Research & Education Initiative, University of Iowa, Iowa City, IA 52242, USA; 4 Iowa City VA Health Care System, Iowa City, IA 52246, USA

**Keywords:** metabolic dysfunction-associated steatotic liver disease (MASLD), metabolic dysfunction-associated steatohepatitis (MASH), obesity, bariatric surgery, endoscopic bariatric and metabolic therapies

## Abstract

This review examines the effectiveness of various surgical and endoscopic bariatric interventions in improving several components of metabolic dysfunction-associated steatotic liver disease (MASLD). Roux-en-Y gastric bypass (RYGB) consistently showed substantial long-term reductions in liver fat, inflammation, and fibrosis, achieving resolution of steatosis in up to 95% of cases. Vertical sleeve gastrectomy (VSG) provided comparable hepatic benefits, significantly reducing liver fibrosis and steatosis in approximately 60% of patients. Adjustable gastric banding (AGB) demonstrated meaningful though comparatively modest hepatic improvements, with steatosis resolution in about 42% of patients. More aggressive procedures like biliopancreatic diversion with duodenal switch (BPD-DS) showed profound metabolic effects, though with increased nutritional risk. Endoscopic therapies, including intragastric balloon (IGB) and endoscopic sleeve gastroplasty (ESG), offered notable short- to medium-term hepatic improvements, significantly reducing steatosis and fibrosis markers. Newer therapies like duodenal mucosal resurfacing (DMR) and the duodenal-jejunal bypass liner showed promising preliminary results, warranting further investigation. Overall, surgical interventions remain superior for sustained liver health improvements, while endoscopic therapies present viable alternatives for patients requiring less invasive interventions.

## 1. Introduction

Obesity is a growing global health crisis. According to the World Health Organization (WHO), in 2022, 2.5 billion adults (43% of the population) were overweight, with 890 million classified as obese [1]. Obesity is a major risk factor for numerous comorbidities [2], including cardiovascular disease [3], type 2 diabetes mellitus (T2DM) [4], hypertension [5], malignancies [6], and non-alcoholic fatty liver disease (NAFLD) [2,7]. NAFLD, characterized by excessive hepatic fat accumulation independent of alcohol consumption, affects approximately 25–38% of the global population [8] and is now the leading cause of chronic liver disease in many countries [9]. It may progress to non-alcoholic steatohepatitis (NASH), which affects ~60% of NAFLD patients and can advance to fibrosis, cirrhosis, or hepatocellular carcinoma [9]. Although NAFLD is strongly associated with obesity, present in about 50% of cases and ~80% of NASH cases [9], disease severity does not always correlate with body weight [10]. These alarming trends highlight the need for effective interventions. Lifestyle modification, including diet and exercise, often leads to only modest and unsustained weight loss [11], and pharmacologic treatments offer moderate benefit with frequent side effects [12]. Bariatric surgery remains the most effective long-term therapy for severe obesity. Procedures like Roux-en-Y gastric bypass (RYGB) and vertical sleeve gastrectomy (VSG) are associated with significant metabolic improvements and reductions in NAFLD severity [13,14,15], including reductions in liver fat, liver enzymes, and fibrosis [16,17,18]. However, limited access and surgical risks have led to increased interest in endoscopic bariatric and metabolic therapies (EBMTs) such as endoscopic sleeve gastroplasty (ESG) and intragastric balloon (IGB) placement [19,20]. These less invasive options offer lower risk, quicker recovery, and promising results in liver fat reduction, enzyme improvement, and fibrosis attenuation [21,22]. This review explores the impact of bariatric and endoscopic interventions on MASLD and MASH, recently redefined from NAFLD and NASH, respectively.

## 2. Methods

A systematic search strategy was employed using standard databases including PubMed, MEDLINE, and Google Scholar to identify relevant studies on bariatric surgical and endoscopic interventions and their impacts on metabolic dysfunction-associated steatotic liver disease (MASLD). Keywords such as “MASLD”, “MASH”, “NASLD”, “NASH”, “obesity”, “bariatric surgery”, “Roux-en-Y gastric bypass”, “sleeve gastrectomy”, “endoscopic sleeve gastroplasty”, “intragastric balloon”, and related terms were utilized. Articles selected included randomized controlled trials, prospective and retrospective cohort studies, and meta-analyses. Inclusion criteria encompassed studies examining liver histology, biochemical markers, liver enzymes, and non-invasive liver health assessments post-intervention. The data extracted focused on clinical outcomes, safety profiles, and procedural effectiveness concerning hepatic improvement. The quality and relevance of the included studies were critically appraised based on standardized methodological criteria. Extracted data were then synthesized and organized thematically by procedure type, intervention outcomes, and time frame of observed liver health improvements. The quality of studies was assessed using the Newcastle–Ottawa Scale for observational studies and the Cochrane Risk of Bias tool for randomized controlled trials.

## 3. Results

### 3.1. Roux-en-Y Gastric Bypass (RYGB)

RYGB, first introduced by Mason in 1966, has since become a cornerstone bariatric procedure recognized for its substantial and sustained effectiveness [23,24]. The surgical technique entails creating a small gastric pouch and rerouting a segment of the small intestine, leading to notable reductions in food intake and nutrient absorption. Patients undergoing RYGB typically achieve a significant weight loss of approximately 60–70% of their excess body weight, accompanied by marked improvements in obesity-associated comorbidities, including type 2 diabetes mellitus (T2DM), hypertension, and hyperlipidemia [23]. Metabolic and bariatric surgery (MBS), including RYGB, is currently recommended for individuals with a body mass index (BMI) greater than 35 kg/m^2^ irrespective of comorbidities, and also for patients with a BMI between 30 and 34.9 kg/m^2^ who present with metabolic disease. For Asian populations, the threshold is lower, with MBS indicated for a BMI greater than 27.5 kg/m^2^ [13,25]. A comprehensive systematic review involving 8818 patients demonstrated that the pooled mean total weight loss (%TWL) following RYGB reached 31.9% after one year [26]. RYGB has a particularly profound impact on liver health, notably in individuals with metabolic dysfunction-associated steatotic liver disease. Evidence from recent systematic reviews underscores substantial improvements in liver histology post-RYGB, including significant reductions in fibrosis and hepatic steatosis. Specifically, de Brito e Silva et al. reported a 26% overall reduction in liver fibrosis severity and a 53% decrease in steatohepatitis activity, with notable improvements in the NAFLD activity score (NAS) [27]. In a landmark prospective trial, Verrastro and colleagues demonstrated that one year following RYGB surgery, resolution of non-alcoholic steatohepatitis (NASH) without progression of fibrosis occurred in 56% of patients compared to only 16% in patients managed by lifestyle modifications alone (*p* < 0.0001) [28]. Furthermore, longitudinal data indicate continued improvement in hepatic outcomes beyond the initial postoperative period. Studies following patients up to five years post-RYGB report persistent and progressive reductions in hepatic steatosis, inflammation, and fibrosis. According to a study by Fakhry et al., significant improvements in liver histology were observed as early as six months post-surgery, with sustained resolution or marked reduction in NAFLD in approximately 70–85% of patients over a five-year period [29]. Additionally, long-term follow-up data have demonstrated reduced incidence of progression to advanced liver disease such as fibrosis in individuals undergoing RYGB compared to nonsurgical treatments, reinforcing its critical therapeutic role in managing NAFLD [30]. Prospective studies further emphasize the benefits of RYGB on NAFLD. Cazzo et al. demonstrated a significant reduction in NAFLD fibrosis scores within 12 months post-surgery, emphasizing the procedure’s capacity to reverse early liver damage associated with obesity [30]. Similarly, Moretto et al. observed substantial histological improvements in liver fibrosis, with over 70% of patients showing regression in fibrosis severity one year after RYGB surgery [31]. Histological evaluation by Mottin et al. reported that bariatric surgery-induced weight loss led to marked improvement or resolution of hepatic steatosis in morbidly obese patients, evident within the first postoperative year [32]. Barker et al. further confirmed these benefits, demonstrating significant regression in hepatic inflammation and fibrosis scores in patients with NASH at 21.4 months post-RYGB surgery [33]. Additionally, recent long-term follow-up data from Norouzian Ostad et al. showed sustained reductions in hepatic fibrosis up to 30 months after RYGB, reinforcing the procedure’s lasting beneficial effects on liver health [34]. These findings collectively highlight RYGB not only as a powerful strategy for substantial weight loss but also as a highly effective therapeutic intervention for MASLD, underscoring its potential to mitigate severe hepatic outcomes and significantly enhance overall metabolic health.

### 3.2. Vertical Sleeve Gastrectomy (VSG)

VSG, initially introduced in 1988 as a component of biliopancreatic diversion with duodenal switch (BPD-DS), involves a subtotal resection of the gastric fundus and body to create a tubular gastric sleeve, significantly reducing stomach volume [35]. The resulting weight loss from VSG is attributed to gastric restriction, neurohormonal modulation, and altered gastric emptying [36]. A comprehensive systematic review involving 3542 patients documented a pooled mean total weight loss (%TWL) of approximately 29.5% at one year post-surgery [26]. Growing evidence strongly supports VSG’s beneficial impact on liver health, particularly regarding MASLD. Heilberger et al. found notable reductions in liver fibrosis scores, including aspartate aminotransferase to platelet ratio (APRI), Forns, and NAFLD-specific scores, three years following VSG, although improvements in FIB-4, BARD, and LOK scores were comparatively modest [37]. Additionally, a retrospective analysis by Algooneh et al. reported remission of NAFLD in 56% of patients undergoing VSG, with a highly significant association between NAFLD remission and achieving greater than 50% excess weight loss (odds ratio 10.1, *p* < 0.001), further stressing the relationship between effective weight loss and liver disease resolution [38]. Studies employing advanced imaging and diagnostic techniques further affirm the positive hepatic outcomes following VSG. Batman et al. demonstrated significant reductions in liver stiffness and controlled attenuation parameter (CAP) values, indicative of improved hepatic steatosis and fibrosis, as early as six months post-VSG [39]. Similarly, Chen et al. utilized computed tomography-based gastric volumetry, revealing substantial decreases in hepatic fat content correlating with significant weight reduction at one year after surgery [40]. Histological data from biopsy-proven NAFLD cases further reinforce these findings. Cherla et al. reported significant improvements in liver histology post-VSG, with marked reductions in both steatosis and fibrosis evident by 12 months postoperatively [41]. Collectively, these studies indicate that VSG effectively ameliorates hepatic fibrosis and steatosis associated with MASLD. Typically, around 50–60% of patients experience substantial histological and clinical improvement or resolution of MASLD within one to three years postoperatively. These findings underscore VSG’s important role in managing MASLD, positioning it as a viable surgical option to significantly improve liver health outcomes alongside weight loss.

### 3.3. Adjustable Gastric Banding (AGB)

AGB was introduced in the 1990s as an alternative to restrictive bariatric procedures and has proved capable of substantial and sustained weight reduction with a low perioperative risk; it has also demonstrated a significant therapeutic impact on NAFLD by inducing weight loss that translates into histopathologic improvements of steatosis and improvement of NASH [42,43,44]. In a seminal biopsy study by Dixon et al., 36 adults underwent paired liver biopsies at ~2 years after AGB: nearly all showed major reductions in lobular steatosis, inflammation, and fibrosis (*p* < 0.001). The proportion meeting NASH criteria fell from 64% pre-operatively to ~11% on follow-up, and the number of patients with significant fibrosis (stage ≥ 2) dropped from 18 to three (*p* < 0.001) [45]. A subsequent cohort of 60 AGB patients with ~30-month liver biopsies confirmed these findings, with only 10% having persistent NASH postoperatively versus 50% at baseline. On average, ~30–34 kg of weight loss (~50% excess weight) was achieved in these series, and those with metabolic syndrome experienced the greatest histologic gains [46]. Notably, fibrosis regressed alongside steatosis resolution in many patients, indicating partial reversal of liver injury with AGB-induced weight reduction. Biochemical improvements paralleled histology: for instance, declines in serum γ-glutamyl transferase (GGT) strongly correlated with and predicted improvements in inflammation, fibrosis, and overall NAFLD activity score [46]. Improvements in hepatic fat are evident even within months of surgery—Phillips et al. reported significant decreases in liver fat fraction by 3 months in non-diabetic women post-AGB (using proton magnetic resonance spectroscopy, *p* < 0.05)—suggesting early resolution of steatosis [47]. Importantly, the liver benefits of AGB extend to younger populations. Adolescent bariatric series have shown that AGB can safely ameliorate pediatric NASLD. For example, Loy et al. followed 56 severely obese adolescents with fatty liver evidence through 1–2 years after AGB: the cohort’s NAFLD severity scores fell significantly within one year (mean decrease ~0.7 points, *p* < 0.01), with further improvement by two years. This was accompanied by a profound reduction in metabolic syndrome prevalence from 27% pre-surgery to just 2% at 24-month follow-up, underscoring concurrent metabolic and hepatic benefits [48]. Such pediatric findings are consistent with earlier reports of AGB-driven weight loss improving steatosis in younger patients and mirror the robust histological improvements seen in adults. In summary, AGB-induced weight loss confers meaningful therapeutic effects on MAFLD achieving resolution of hepatic steatosis and substantial improvements in necroinflammation and fibrosis, thereby markedly improving liver histology and metabolic health in obese individuals [46]. The evidence to date positions AGB as an effective intervention for obesity-related fatty liver disease, capable of reversing key features of MASLD and mitigating progression to advanced fibrosis.

### 3.4. Biliopancreatic Diversion with Duodenal Switch (BPD-DS)

BPD-DS, developed by Dr. Scopinaro in 1979, involves partial gastrectomy and rerouting of the intestines to limit nutrient absorption. Modifications to the procedure have improved safety and outcomes [49]. It is a combined restrictive and malabsorptive bariatric procedure and has shown profound therapeutic effects on metabolic dysfunction-associated steatotic liver disease in patients with severe obesity [35]. BPD-DS induces greater and more sustained weight loss than other bariatric surgeries, with excess weight loss often ~80% at 2 years in morbidly obese patients [50]. This marked weight reduction, along with hormonal and metabolic alterations, directly targets hepatic steatosis and insulin resistance—the crux of NAFLD [51]. Histologically, BPD-DS yields significant improvements: Keshishian et al. reported a ~60% reduction in hepatic steatosis accompanied by a three-grade improvement in NASH severity within 3 years postoperatively [52]. By 12 months, transient early elevations in ALT and AST fully normalize and no lasting hepatic functional impairment is observed [52]. Accordingly, BPD-DS leads to resolution of steatohepatitis in a majority of patients and can even regress advanced disease [53,54,55]. For example, one five-year follow-up study on 37 patients with biopsy-confirmed NASH who underwent BPD-DS found that 26 (70%) achieved NASH resolution, attributing this improvement primarily to reduced insulin resistance rather than weight loss alone [51]. Improvements in liver biochemistry mirror these histologic gains: serum ALT and AST drop markedly after BPD-DS as hepatic inflammation abates (the proportion of patients with elevated ALT fell from 82% pre-surgery to 20% at 4 years post-BPD, and AST from 70% to 6%) [56]. BPD-DS also rapidly enhances insulin sensitivity—HOMA-IR improves within days postoperatively [57], and it achieves high rates of type 2 diabetes remission, addressing a key driver of NAFLD [51,54]. Notably, fibrosis outcomes after BPD-DS can be heterogeneous; Russo et al. demonstrated fibrosis reversal in 16% of patients (*p* = 0.022) and improvement in 32% [51]. Additionally, Weiner et al. had shown that ten patients out of twelve (83%) achieved complete remission in fibrosis, and the remaining two showed improvements [54]. However, one series found fibrosis scores worsened in 40% of patients (improved in 27%) despite reduced steatosis [53]. Proposed mechanisms for such paradoxical fibrosis progression include the effects of rapid weight loss, bacterial overgrowth, and deficiencies in protein or micronutrients [58,59]. Indeed, BPD-DS carries well-recognized risks: protein–calorie malnutrition and fat-soluble vitamin deficiencies occur in a subset, and rare cases of acute liver failure have been documented, specifically after this procedure and some patients with malabsorptive complications have required revisional surgery (lengthening of the common channel or conversion to a gastric bypass) to halt progressive liver dysfunction [60]. BPD-DS achieves superior weight loss compared to RYGB, with mean BMI reductions of ~24.8 kg/m^2^ versus ~17.3 kg/m^2^, respectively (*p* < 0.001) [54,61,62,63]. However, its complexity, risks of malabsorption and its nutritional consequences, and the rare potential for accelerated liver fibrosis limit widespread adoption [53,58,59,60].

### 3.5. Intragastric Balloon (IGB)

IGB therapy represents a short-term, minimally invasive intervention for weight loss in obese individuals, typically promoting a total body weight loss (TBWL) ranging from 7.5% to 14% [64,65,66,67]. Initially introduced in 1984, the first FDA-approved balloon, the Garren-Edwards Gastric Bubble (GEGB), was discontinued after seven years due to significant adverse effects, including gastric erosion, ulcers, small bowel obstruction, and inadequate weight reduction [68,69]. Modern advancements have led to safer and more effective balloon systems, predominantly saline- or air-filled, that function by occupying gastric space to enhance satiety, generally achieved with volumes of approximately 400 mL or greater [64]. In the United States, IGB use is indicated for patients with a BMI between 30–35 kg/m^2^, while European guidelines extend usage to those with BMIs between 27–35 kg/m^2^ [69]. Currently approved devices include Obalon, Orbera, and ReShape in the U.S., alongside CE-marked alternatives such as Elipse and Spatz3 [70]. IGB therapy notably benefits hepatic health, especially in NAFLD and NASH management. A meta-analysis involving six studies reported an 83.5% improvement in NAFLD activity score (NAS) following IGB therapy [71]. Complementary evidence from a systematic review highlighted a significant histological improvement, demonstrating a reduction in NAS scores from 4 ± 2.25 to 2 ± 0.75 (*p* = 0.03) within six months [72]. Further supporting this, a cohort study employing magnetic resonance elastography and liver biopsies found an 87% patient cohort experienced a median NAS reduction of three points, with 73% achieving reductions of at least two points [73]. IGB therapy also robustly decreases hepatic steatosis; hepatic fat improvement was reported in 79.2% of patients following balloon placement [71], and studies utilizing ultrasound and MRI observed hepatic fat fraction reductions from 16.7 ± 10.9 to 7.6 ± 9.8 (*p* = 0.003) [72]. Prospective findings further corroborated these results, documenting severe hepatic steatosis decline from 52% pre-treatment to just 4% after six months (*p* < 0.0001) [74]. Liver function markers also significantly improved; meta-analyses showed substantial decreases in ALT (−9 U/L) and AST (−3 U/L) levels [75]. Consistently, a prospective evaluation demonstrated abnormal ALT levels dropping from 38% to 7% at six months [74], and another study reported reductions in ALT and GGT from 42% to 22% and 57% to 34%, respectively, signifying enhanced liver function particularly in metabolically compromised patients [76]. Overall, the accumulated evidence positions IGB therapy as an effective approach for hepatic fat reduction, liver function enhancement, and significant histological improvement in MASLD and MASH. In addition, the TransPyloric Shuttle (TPS) represents a novel non-invasive silicone device designed similarly for weight management. TPS comprises a larger spherical bulb tethered to a smaller cylindrical one, which is endoscopically positioned across the pylorus to delay gastric emptying and increase satiety through intermittent occlusion [77]. FDA-approved for treatment durations of up to 12 months, TPS demonstrated notable effectiveness in the ENDObesity trial—an open-label study involving 20 patients with BMIs between 30–40 kg/m^2^ and comorbid conditions including diabetes—yielding significant weight reduction [77]. A subsequent randomized trial combining TPS with lifestyle modifications reported an average TWL of 9.5% after one year [78]. Nevertheless, existing research concerning TPS’s specific effects on MASLD and MASH remains limited, underscoring a critical need for further targeted investigations into its hepatic benefits.

### 3.6. Endoscopic Sleeve Gastroplasty (ESG)

ESG, first performed in 2012, is a minimally invasive weight loss procedure that reduces gastric volume using full-thickness endoluminal sutures. ESG mimics aspects of sleeve gastrectomy but without surgical incisions. Advances in suture design, such as the ‘U’ stitch, have improved durability and outcomes [79,80,81]. ESG has demonstrated significant benefits for NAFLD and NASH. A systematic review and meta-analysis involving 175 patients found ESG significantly reduced the NAFLD fibrosis score by 0.5 points and hepatic steatosis index by 4.85 points. Patients also experienced ALT reductions of 6.32 U/L and a total weight loss of 17.28% [82]. A 26-patient study confirmed reductions in ALT, hepatic steatosis index, and NAFLD fibrosis score at 6 and 12 months post-procedure [83]. A randomized controlled trial comparing ESG plus lifestyle modifications with a sham endoscopy group found ESG significantly reduced liver stiffness (*p* < 0.05) [84]. Additionally, 20% of participants with advanced fibrosis (F3–F4) improved to lower fibrosis stages (F0–F2) after ESG [85]. Long-term studies indicate ESG continues to benefit liver health. A longitudinal study found that patients maintained reductions in NAFLD fibrosis score and hepatic steatosis index, decreasing by 0.3 and 4 points per year, respectively [85]. Another meta-analysis confirmed improved metabolic markers and liver function 12 months post-ESG [82]. In conclusion, ESG provides significant improvements in MASLD and MASH, along with substantial weight loss. However, further studies with larger sample sizes and longer follow-ups are needed to validate its sustained impact on liver disease. These findings highlight the potential of EBMTs in mitigating MASLD and MASH, offering less invasive, effective alternatives to bariatric surgery for patients with obesity-related liver disease.

### 3.7. Primary Obesity Surgery Endolumenal (POSE)

POSE utilizes an incisionless operating platform (IOP; USGI Medical, San Clemente, CA, USA) to create full-thickness gastric plications for weight loss. The system includes the g-Cath Suture Anchor Delivery Catheter and accessories such as the Transport (a steerable, multi-lumen access device) and endoscopic graspers for tissue manipulation. Typically, 7–9 suture anchors reduce fundal volume and compliance, while an additional 3–4 anchors in the distal body induce antral dysmotility, enhancing satiety [86]. Due to a high incidence of adverse effects, POSE was modified into POSE 2.0, which focuses on the gastric body with multiple suture plications to enhance motility and reduce gastric size more effectively [87]. A clinical trial involving 42 adults (20 POSE 2.0 and 22 control) found that POSE 2.0 significantly improved hepatic steatosis and liver enzyme levels over 12 months compared to lifestyle modifications alone [88]. Notably, 31.6% of POSE 2.0 patients achieved complete steatosis resolution at 6 months, increasing to 52.6% at 12 months, while no improvements were observed in the control group. Liver function markers, including the hepatic steatosis index (HSI) and aspartate aminotransferase to platelet ratio (APRI), also showed significant improvement. POSE 2.0 patients experienced an average TBWL of 18.0% at 6 months and 17.5% at 12 months [88]. Despite these promising results, additional studies are needed to confirm POSE’s long-term efficacy in managing MASLD.

### 3.8. Aspiration Therapy

AspireAssist (Aspire Bariatrics, King of Prussia, PA, USA) is an endoscopic device for aspiration therapy, designed to remove approximately 30% of ingested calories from the stomach post-meal. Approved by the FDA in 2016 for patients with BMIs of 35–55 kg/m^2^, it consists of a 30-French aspiration tube inserted endoscopically using a standard pull technique, connected externally to a portable aspiration device [89,90]. A systematic review of 37 trials involving 15,639 patients found that aspiration therapy resulted in a significantly higher TBWL (12.1–18.3%) compared to lifestyle modifications alone (3.5–5.9%) at 12 months [66]. Additionally, a meta-analysis of 590 patients reported significant reductions in ALT (−5.2 to −9.8 U/L) and AST (−2.7 U/L) levels (*p* < 0.0001) [91]. While these findings suggest potential liver benefits, further research is required to assess the long-term effects of AspireAssist on MASLD and MASH.

### 3.9. Duodenal Mucosal Resurfacing (DMR)

DMR is an endoscopic metabolic therapy first evaluated in 2016 for patients with type 2 diabetes mellitus (T2DM). The procedure involves circumferential ablation of the duodenal mucosa, extending from the ampulla of Vater to the ligament of Treitz [92]. While weight loss following DMR is modest (~2.5 kg at six months), significant metabolic benefits have been reported. Improvements in glycemic control, including a 1.2% reduction in HbA1c at six months, have been observed, particularly following long-segment ablation [92]. Beyond glycemic improvements, DMR has shown potential hepatic benefits. The REVITA-2 study assessed liver fat using the MRI-proton density fat fraction (MRI-PDFF) and found a significant reduction in liver fat content three months post-DMR in patients with NAFLD, compared to sham procedures [93]. At six months, ALT levels decreased by 29.3% with concurrent FIB-4 score improvements [94]. Additionally, DMR exhibited insulin-sensitizing and lipid-lowering effects independent of weight loss or glycemic control, making it a promising intervention for MASLD and MASH. Further studies are needed to establish its long-term role in metabolic and liver disease management.

### 3.10. Duodenal-Jejunal Bypass (DJB) Liner or Sleeve

DJBL, commercially known as EndoBarrier™, is a 60 cm impermeable sleeve inserted endoscopically into the duodenum to reduce nutrient absorption, mimicking the effects of Roux-en-Y gastric bypass [95,96]. Early trials reported significant weight loss within 12 weeks, with some patients achieving up to 12% TBWL [95]. However, a U.S. trial (2012–2016) was halted due to a higher-than-expected incidence of hepatic abscesses (3.3%) [97]. Despite this, international data have not demonstrated similar safety concerns [98]. DJBL has demonstrated metabolic benefits, including weight loss and glycemic improvements. A German cohort study found a median weight loss of 10% and a BMI reduction from 41 kg/m^2^ to 38 kg/m^2^ (*p* < 0.01) after nearly one year of DJBL use [98]. Another study reported a TWL of 9.1% during treatment, with patients maintaining a 2.2% TWL at four-year follow-up [99]. A Chinese study on the TONGEE DJBS device reported an 8.9% TWL in three months, with over 50% of patients maintaining > 5% TWL six months post-removal [100]. DJBL has also shown promise in MASLD and MASH treatment. A study using FibroScan demonstrated reduced hepatic steatosis, with CAP scores decreasing from 343 dB/m to 317 dB/m (*p* < 0.05) and liver stiffness improving from 10.4 kPa to 5.3 kPa (*p* < 0.01) after one year [98]. A separate study reported a CAP reduction from 312.7 to 274.0 dB/m (*p* < 0.001) after three months of DJBL therapy, with 42% maintaining lower steatosis scores six months post-explant [100]. While DJBL appears to improve hepatic steatosis and metabolic markers, its effects on fibrosis remain inconclusive [101,102,103]. Notably, DJBL use has been associated with malabsorption-related deficiencies in ferritin, albumin, vitamin B12, and folic acid [104]. The FDA has approved a new DJBL trial, “RESET”, expected to conclude in 2026 [105]. These findings highlight the potential of emerging endoscopic bariatric therapies in managing obesity-related liver disease. However, further large-scale, long-term studies are necessary to validate their efficacy and safety for MASLD and MASH treatment.

### 3.11. Magnetic Anastomosis System (MAS)

The Magnetic Anastomosis System (MAS) is an innovative endoscopic bariatric procedure that enables the formation of an anastomosis between two segments of the small intestine without the need for surgical incision. Early studies, including preclinical research on porcine models, have demonstrated the safety and efficacy of this technique [106]. However, clinical data remain limited, with procedural variations across studies. A notable study involving eight patients introduced a novel approach in which a duodenal magnet was inserted endoscopically while an ileal magnet was placed laparoscopically. The magnets were aligned to compress the intervening tissue, leading to tissue necrosis and subsequent anastomosis formation. The magnets were naturally expelled within approximately 30 days. A one-year follow-up revealed a patent anastomosis, with 87.5% of patients achieving > 5% TWL and 75% maintaining HbA1c levels below 7% [107]. A recent study combined MAS with vertical sleeve gastrectomy (VSG) using a modified magnetic system (GT Metabolic Solutions, San Jose, CA, USA). This method eliminated the need for an open gastrointestinal tract incision, as both magnets were placed endoscopically and positioned laparoscopically. The procedure resulted in a 100% patent anastomosis with no leaks or serious adverse effects. At 12-month follow-up, patients who underwent simultaneous magnet placement with VSG achieved 34.0% TWL, while those who received MAS after VSG showed an 11.0% TWL. Both groups demonstrated significant HbA1c reductions at six months, with all patients reducing their need for antidiabetic medications [108]. A recently completed clinical trial involving 10 patients reported a significant decrease in BMI, from 44.08 ± 3.29 kg/m^2^ to 28.69 ± 3.9 kg/m^2^ at one year (*p* < 0.001). Additionally, all patients discontinued their antihypertensive, antidiabetic, and statin medications post-procedure [109]. MAS represents a promising minimally invasive bariatric technique with potential metabolic benefits. However, the current evidence is limited by small sample sizes and the absence of control groups. Large-scale randomized controlled trials are needed to validate its long-term efficacy, particularly regarding its impact on liver function, MASLD, MASH, and fibrosis progression.

## 4. Discussion

This review evaluates various bariatric and metabolic interventions based on their impact on metabolic dysfunction-associated steatotic liver disease (MASLD) and metabolic dysfunction-associated steatohepatitis (MASH). The procedures examined include surgical approaches such as Roux-en-Y gastric bypass (RYGB), vertical sleeve gastrectomy (VSG), adjustable gastric banding (AGB), and biliopancreatic diversion (BPD), as well as endoscopic techniques, including intragastric balloon (IGB), endoscopic sleeve gastroplasty (ESG), primary obesity surgery endolumenal (POSE), aspiration therapy, duodenal mucosal resurfacing (DMR), and the duodenal-jejunal bypass liner (EndoBarrier). Comparison of these interventions based on their effects on liver enzymes, biopsy findings, NAS score, steatosis, and liver fibrosis (using non-invasive imaging techniques) shows the following:

### 4.1. Liver Enzyme Improvement

Several surgical procedures initially cause transient increases in ALT and AST levels postoperatively, followed by substantial reductions over time. By six months, VSG exhibited the greatest reduction in ALT levels (50%), decreasing from 31.4 IU/L to 15.7 IU/L [39]. EndoBarrier therapy followed with a 48% reduction (54 IU/L to 28 IU/L) [96], and RYGB achieved a 28.5% decrease [110]. Other procedures showed variable ALT reductions: IGB (20.3–38%) [74,76], DMR (29.3%) [94], and ESG (16%) [83]. At one-year follow-up, BPD-DS and RYGB demonstrated sustained improvements, with ALT reductions of 46% and 45%, respectively. RYGB continued to show progressive benefits, with ALT declining by 61.3% over three years [111]. In contrast, VSG outperformed RYGB at two years, achieving a 32.1% reduction compared to 15.4% with RYGB [112,113]. Aspiration therapy, though effective, resulted in slower ALT declines of 5.2–8.9 U/L over 12 months [91]. This suggests that while surgical procedures provide long-term benefits, non-invasive therapies offer gradual but meaningful improvements.

### 4.2. Fibrosis and Steatosis Improvement

Surgical procedures consistently led to reductions in steatosis, NAS, and fibrosis, though the degree and timing of improvement varied. RYGB achieved steatosis remission in 89–95% of patients, especially in those with moderate-to-severe baseline steatosis (grades 2 and 3) [114,115,116,117]. VSG showed similar efficacy, with 56–68.4% achieving remission [38,118]. BPD-DS showed slower but sustained benefits, reducing steatosis by 35% at one year and 60% by year three [52]. AGB also improved steatosis (41.9%), although RYGB showed greater efficacy (76.2%) [119]. IGB rapidly reduced steatosis from 52% to 4% within six months, as measured by ultrasonography [74], supporting its short-term potential. RYGB also reduced MASH severity by 75–88.9%, with 38% of patients achieving full remission [114,116], while VSG achieved 100% resolution [118]. BPD-DS showed delayed but substantial improvement, including a three-grade reduction in MASH over three years [52] AGB showed modest NAS improvement (35.3%) [119]. A multicenter study of 284 patients found MASLD severity improved in 93.7% overall—highest with BPD-DS (100%), followed by RYGB (88.2%) and AGB (61.9%) [54].

*FibroScan (transient elastography) findings*: VSG produced the largest CAP score reduction (91.8 dB/m) [39], while ESG had a more modest decrease (41.12 ± 49.41 dB/m; *p* = 0.067) [84]. EndoBarrier and DJBS each reduced CAP by 26–42 dB/m (*p* < 0.001 and *p* < 0.005, respectively) [98,103]. POSE showed the most significant improvement among endoscopic therapies, with an 87 dB/m drop at 12 months (*p* < 0.001) [88].

*Hepatic steatosis index (HSI):* EndoBarrier reduced HSI by five points at 9 months (*p* < 0.001) [100], POSE by 11.16 points (*p* < 0.002) [88], and ESG by 4–4.85 points at one year [82,85].

*NAFLD activity score (NAS):* IGB reduced NAS by two points in six months, with 87% of patients achieving ≥ 3-point improvement [72,73]. ESG showed a four-point drop in patients with >10% weight loss [84]. RYGB and VSG achieved 2.8- and 2.3-point reductions, respectively [120].

*Fibrosis improvement:* EndoBarrier lowered NFS by one point (*p* < 0.05), with sustained post-removal benefits [102]. RYGB and VSG improved fibrosis scores by 0.9 and 0.65 points, respectively [121,122]. ESG led to a 0.5-point NFS drop (*p* < 0.001) [82], while BPD reduced NFS by 1.5 points, though FIB-4 remained unchanged [51].

*Liver stiffness:* EndoBarrier reduced liver stiffness by 5.1 kPa within three months [98]. VSG lowered it from 7.5 to 5.6 kPa in six months [39]. ESG showed gradual improvements [84], while POSE did not significantly change fibrosis [88]. APRI scores improved with EndoBarrier (45–55%) [102], POSE (31%) [68], and IGB (37.8%) [73].

Comparison of variable MASLD endpoints based on procedural intervention shows the following:

### 4.3. Bariatric Surgeries

Surgical bariatric interventions, especially Roux-en-Y gastric bypass (RYGB), demonstrated significant and sustained improvements in hepatic outcomes, substantially reducing hepatic steatosis and fibrosis through long-term follow-up. RYGB’s effectiveness is attributed to marked weight loss combined with profound metabolic and hormonal changes that collectively enhance liver health beyond mere caloric restriction. Vertical sleeve gastrectomy (VSG) similarly offered substantial hepatic benefits, although slightly less robust, significantly reducing hepatic steatosis and fibrosis in about 60% of patients, primarily due to restrictive mechanisms and hormonal shifts, and notably increased GLP-1 secretion. Adjustable gastric banding (AGB) yielded modest hepatic improvements, reflecting its more gradual and less substantial weight loss, leading to lower resolution rates of steatosis. Biliopancreatic diversion with duodenal switch (BPD-DS) provided significant hepatic and metabolic benefits through pronounced malabsorption and hormonal alterations; however, these advantages were tempered by increased nutritional and hepatic risks. Overall, surgical interventions demonstrated significant steatosis resolution, particularly notable with RYGB (~90–95%) and VSG (~60%), moderate with AGB (~40%), and substantial with BPD-DS. Similarly, fibrosis improvements were robust with RYGB and VSG, variable but significant with BPD-DS, and modest with AGB (see Table 1).

### 4.4. Bariatric Endoscopy

Endoscopic interventions, including intragastric balloon (IGB) and endoscopic sleeve gastroplasty (ESG), demonstrated notable improvements in hepatic health over the short-to-medium term. IGB provided rapid reductions in hepatic steatosis and fibrosis, making it particularly suitable for patients requiring immediate metabolic enhancements. ESG similarly delivered significant hepatic benefits, effectively reducing liver fat and enhancing fibrosis markers. Additionally, emerging techniques such as duodenal mucosal resurfacing (DMR) and duodenal-jejunal bypass liners (DJBLs) have shown promising preliminary outcomes, significantly decreasing hepatic steatosis and improving biochemical and fibrosis indicators. Although these results are encouraging, the existing evidence remains limited, highlighting the necessity for longer-term and larger-scale studies to fully establish these procedures’ roles in clinical practice. Overall, endoscopic therapies exhibited rapid and substantial reductions in steatosis, especially evident with IGB and ESG, alongside promising preliminary results for DMR and DJBL. Fibrosis improvements across these methods were mild to moderate but clinically meaningful, underscoring the need for further longitudinal research (see Table 2).

## 5. Conclusions

This comprehensive analysis underscores the differential efficacy of surgical versus endoscopic bariatric procedures in managing MASLD. Surgical procedures, especially RYGB, offer robust, durable hepatic improvements, while endoscopic procedures provide effective short-to-medium-term alternatives, particularly for patients seeking less invasive treatments. Optimal procedural selection should consider patient-specific clinical factors, hepatic severity, and long-term therapeutic objectives. Continued research through well-designed longitudinal studies will be critical to refine and optimize MASLD management strategies.

## Figures and Tables

**Table 1:** Effect of bariatric surgery on MASLD—comparing RYGB, VSG, AGB, and BPD-DS across key clinical endpoints (steatosis, fibrosis, safety, weight loss, metabolic outcomes).

Refs.	Citation	Intervention	Study Type	Duration	Diagnosis Method	Effect on Steatosis	Effect on Fibrosis	Effect on Liver Enzymes
1	Mottin et al. [32]	RYGB	Retrospective cohort study	12 months	Liver biopsy	Steatosis regressed in 54.4% of patients and improved in 27.8% (*p* < 0.0001)		
2	Cazzo et al. [30]	RYGB	Prospective cohort study	12 months	Liver biopsy, NFS		NFS decreased by a mean of 1.08 points at follow-up (*p* = 0.039); 55% had regression of severe fibrosis	ALT levels decreased by 32.6% (*p* < 0.0001), and AST levels decreased by 17.5% (*p* < 0.0005)
3	Norouzian Ostad et al. [34].	RYGB	Prospective cohort study	30 months	Liver biopsy or ultrasound (2D-SWE)		Patients with no fibrosis increased from 55% to 91%, while advanced fibrosis (F4) declined from 21% to 2%	ALT levels decreased by 30.1% (*p* = 0.005); AST levels decreased by 9.2%, (*p* = 0.567); ALP levels increased by 4.6% (*p* < 0.001); GGT levels decreased by 57.6% (*p* = 0.012)
4	Jimenez et al. [121]	RYGB	Prospective cohort study	3 years	NFS		NFS decreased by a mean of 0.90 points at 1 year (*p* < 0.0001) and a mean of 0.50 points from baseline at 3 years (*p* < 0.0001)	ALT levels decreased by 45.9% (*p* < 0.0001), AST levels decreased by 19.2% (*p* = 0.0112)
5	Cherla et al. [41].	RYGB	Retrospective cohort study	4 years	Liver biopsy + liver enzymes			ALT levels decreased by 55.2% after one year (*p* < 0.0001); AST levels decreased by 49.5% after one year (*p* < 0.0001); after one year: 83.9% had normalization of liver enzymes
6	Johansson et al. [111]	RYGB	Prospective cohort study	3 years	Liver enzymes			ALT levels decreased by 45.2% at 1 year (*p* < 0.001), and by 61.3% from baseline at 3 years (*p* < 0.001); GGT levels decreased by 56.9% at 1 year (*p* < 0.001), and by 53.8% from baseline at 3 years (*p* < 0.001)
7	Verrastro et al. [28].	RYGB	Multicenter RCT	12 months	Liver biopsy, NFS, NAS, NASH-CRN system	NAS decreased by 56.2% (*p* < 0.001); NASH resolved in 56% of the patients (*p* < 0.0001)	46% had regression of fibrosis (*p* = 0.017)	ALT levels decreased by 37.41% (*p* <0.0001), AST levels decreased by 22.04% (*p* < 0.0006)
8	de Brito e Silva et al. [27]	RYGB	Review and meta-analysis	14 ± 6 months	Liver biopsy	NAS decreased by 2.52 points (*p* < 0.00001), 53% absolute reduction in the prevalence of steatohepatitis (*p* < 0.00001)	26% absolute reduction in the presence of fibrosis (*p* < 0.0001), with grade of fibrosis improving with a pooled mean reduction of 0.77 points (*p* < 0.00001)	ALT levels decreased by a mean of 12.04 (8.44, 15.65) (*p* < 0.00001); AST levels decreased by a mean of 5.15 (2.39, 7.91) (*p* < 0.0003); ALP levels decreased by a mean of 4.57 (−3.44, 12.59) (*p* = 0.26); GGT levels decreased by a mean of 19.06 (13.77, 24.34) (*p* < 0.0001)
9	Vargas et al. [115]	RYGB	Prospective cohort study	16 months	Liver biopsy	NASH decreased in 84% of patients (*p* < 0.001)	Fibrosis score 0 (normal liver) increased from 3.84% to 34.6%	ALT levels decreased by ~24.5% (*p* = 0.143); AST levels increased slightly by ~2.9% (*p* = 0.862); GGT levels decreased by ~52.2% (*p* < 0.001)
10	Tai et al. [117]	RYGB	Prospective cohort study	12 months	Liver biopsy	Steatosis grade 0 (normal liver) increased from 9.5% of patients to 95.2% (*p* < 0.01)	Fibrosis score 0 (normal liver) increased from 14.3% of patients to 42.9% (*p* < 0.01)	ALT levels decreased by 29.4% (*p* < 0.01); AST levels remained unchanged (27.0 IU/L) (*p* = 0.66); GGT levels decreased by 57.1% (*p* < 0.01)
11	Fakhry et al. [29]	RYGB	Systematic review and meta-analysis	5–60 months	Liver biopsy	Resolution of steatosis was 91% (95% CI: 0.82, −0.97)	31% of patients showed fibrosis improvement (95% CI: 0.17–0.46)	ALT levels decreased by 58% (95% CI: 0.22, 0.94); AST levels decreased by 34% (95% CI: 0.14, 0.54)
12	Caiazzo et al. [119]	RYGB	Prospective cohort study	5 years	Liver biopsy	NAS decreased by 65% at 1 year (*p* < 0.001) and remained reduced by 65% from baseline at 5 years (*p* < 0.001; steatosis decreased by 76% at 1 year (*p* < 0.001) and by 74% from baseline at 5 years (*p* < 0.001)		ALT decreased by 41% at 1 year (*p* < 0.001) and by 39% from baseline at 5 years (*p* < 0.001); AST decreased by 25% at 1 year (*p* = 0.035) and by 11% from baseline at 5 years (*p* = 0.092); GGT decreased by 53% at 1 year (*p* < 0.001) and by 33% from baseline at 5 years (*p* = 0.002); ALP decreased by 9% at 1 year (*p* = 0.035) and by 28% from baseline at 5 years (*p* = 0.003).
13	Furuya et al. [116]	RYGB	Prospective cohort study	2 years	Liver biopsy	Steatosis disappeared in 84% of patients (*p* < 0.05)	75% had regression of fibrosis (*p* < 0.05)	ALT decreased by 22.6% (*p* = 0.081); AST remained stable (*p* = 0.856); ALP decreased by 3.5% (*p* = 0.420); GGT decreased by 52.3% (*p* = 0.000)
14	Schwenger et al. [114]	RYGB	Prospective cohort study	12 months	Liver biopsy	NASH decreased in 89% of patients (*p* < 0.001); NAS decreased by 1.74 points (*p* < 0.001); steatosis grade 0 (normal liver) increased from 14.3% of patients to 80.9% (*p* < 0.001)	Fibrosis score 0 (normal liver) increased from 35.7% of patients to 66.7% (*p* < 0.001)	AST levels decreased by 18.9% (*p* = 0.038); ALT levels decreased by 20.7% (*p* = 0.007); ALP levels slightly increased by 1.6% (*p* = 0.59)
15	Barker et al. [33]	RYGB	Retrospective cohort study	21.4 months (range 13.3–31.7)	Liver biopsy	Steatosis scores decreased in all patients (*p* < 0.001); NASH remitted in 89% of patients	Fibrosis was significantly reduced (*p* = 0.008)	ALT levels increased by 2.7% (*p* = 0.984); AST levels showed no change (*p* = 0.945)
16	Baldwin et al. [120]	RYGB	Systematic review	12–55 months	NAS	NAS decreased by 2.8 points (*p* < 0.0001)	NFS decreased by 1.0 points (*p* < 0.00001)	ALT levels decreased by −12.3 IU/L (*p* < 0.00001), AST levels decreased by −3.6 IU/L (*p* < 0.002)
17	Batman et al. [39]	VSG	Prospective cohort study	6 months	FibroScan (LSM, CAP)	CAP values showed a 29.7% reduction in liver steatosis (from 309.2 to 217.4 dB/m) (*p* = 0.001)	LSM showed a 25.3% decrease in liver stiffness (from 7.5 to 5.6 kPa) (*p* = 0.013)	ALT levels decreased by 50.0% (*p* = 0.001); AST levels decreased by 19.8% (*p* = 0.06); ALP levels decreased by 4.9% (*p* = 0.047); GGT levels decreased by 59.7% (*p* = 0.001)
18	Chen et al. [40]	VSG	Prospective cohort study	12 months	LSDR	LSDR increased by approximately 49.4% (from 0.79 ± 0.26 to 1.18 ± 0.14, *p* < 0.001), fatty liver decreased from 84.1% pre-surgery to 14.3% after SG		ALT levels decreased by 68.2% (*p* < 0.001), and AST levels decreased by 46.7% (*p* < 0.001)
19	Cherla et al. [41].	VSG	Retrospective cohort study	4 years (range: 1–10 years)	Liver biopsy + liver enzymes			ALT levels decreased by 53.8% after one year (*p* < 0.0001); AST levels decreased by 45.4% after one year (*p* < 0.0001); after one year: 76.9% had normalization of liver enzymes
20	Fakhry et al. [29]	VSG	Systematic review and meta-analysis	5–60 months	Liver biopsy			ALT levels decreased by 79% (95% CI: 0.60, 0.97); AST levels decreased by 32% (95% CI: 0.02, 0.67)
21	Alexander et al. [37]	VSG	Retrospective cohort study	36 months	NFS, APRI, FIB-4, BARD, Forns		NFS decreased by 0.82 points at 12 months (from 0.63 to 0.19; *p* < 0.001) and by 1.36 points at 36 months (to 0.73; *p* < 0.001); APRI score decreased by 33.3% at 12 months (from 0.30 to 0.20; *p* < 0.001) and remained reduced at 36 months (to 0.20; *p* < 0.001); FIB-4 score increased by 7.7% at 12 months (from 0.91 to 0.98; *p* < 0.001) and by 23.1% at 36 months (to 1.12; *p* = 0.129); BARD score doubled by 12 months (from 2.00 to 4.00; *p* < 0.001) and remained elevated at 36 months (to 3.00; *p* < 0.001); Forns score decreased by 5.5% at 12 months (from 5.82 to 5.49; *p* < 0.001) and by 9.6% at 36 months (to 5.27; *p* = 0.407); LOK score increased by 77.6% at 12 months (from 1.61 to 0.36; *p* = 0.592) and remained elevated at 36 months (0.60; *p* = 0.782)	
22	Verrastro et al. [28].	VSG	Multicenter RCT	12 months	Liver biopsy, NFS, NAS, NASH-CRN system	NAS decreased by 52 ± 83% (*p* < 0.001), NASH resolved in 57% of patients (*p* < 0.0001)	47% had regression of fibrosis (*p* = 0.017)	ALT levels decreased by 38.7% (*p* < 0.0001), AST levels decreased by 23.6% (*p* < 0.0006)
23	de Brito e Silva et al. [27]	VSG	Review and meta-analysis	14 ± 6 months	Liver biopsy	NAS decreased by 2.25 points (*p* < 0.00001), 42% absolute reduction in the prevalence of steatohepatitis (*p* < 0.00001)	20% absolute reduction in the presence of fibrosis (*p* < 0.05), with grade of fibrosis improving with a pooled mean reduction of 0.76 points (*p* < 0.00001)	ALT levels decreased by a mean of 15.43 (12.99, 17.86) (*p* < 0.00001); AST levels decreased by a mean of 8.02 (5.80, 10.25) (*p* < 0.00001); ALP levels decreased by a mean of 13.75 (3.08, 24.43) (*p* = 0.01); GGT levels decreased by a mean of 12.27 (8.40, 16.15) (*p* < 0.00001)
24	Esquivel et al. [118]	VSG	Prospective cohort study	12 months	Liver biopsy + abdominal US	Steatosis resolved in 93.7% of patients, with 100% improvement of its grade, 100% of patients had resolution of steatohepatitis		
25	Azulai et al. [113]	VSG/RYGB	Retrospective cohort study	2 years	Liver enzymes			Patients with abnormal ALT decreased by 34% in SG and by 11% in RYGB
26	van Berckel et al. [112]	VSG/RYGB	Retrospective cohort study	2 years	Liver enzymes			AST levels decreased by 4.3% after RYGB and by 16.7% after SG (*p* < 0.001); ALT levels decreased by 15.38% after RYGB and by 32.1% after SG (*p* < 0.001); ALP levels increased by 4.9% after RYGB but decreased by 10% after SG (*p* < 0.001)
27	Nickel et al. [122]	VSG + RYGB	Prospective cohort study	12 months	FibroScan, NFS, APRI		LSM showed ~45% decrease in liver stiffness (from 12.9 to 7.1 kPa) (*p* < 0.001); fibrosis stage improved in 94% of patients; patients with advanced fibrosis (F4) dropped from 48% to 16.5%; NFS decreased by 0.68 points (*p* < 0.001), and APRI score decreased by 24.0% (*p* = 0.009)	AST levels decreased by ~32% (*p* < 0.001); ALT levels decreased by ~48% (*p* < 0.001); GGT levels decreased by ~57% (*p* < 0.001); ALP levels decreased ~4.86% (*p* = 0.302)

**Table 2:** Effect of endoscopic bariatric therapies on MASLD—summarizing ESG, IGB, POSE, DMR, DJBL, and other emerging techniques with a similar framework.

**Refs.**	Citation	Intervention	Study Type	Duration	Diagnosis Method	Effect on Steatosis	Effect on Fibrosis	Effect on Liver Enzymes
28	Hajifathalian et al. [85]	ESG	Prospective cohort study	2 years	HSI, NFS	HSI decreased by 4 points per year (*p* < 0.001)	NFS decreased by 0.3 points per year (*p* = 0.034); fibrosis improved from F3–F4 to F0–F2 in 20% of patients	ALT decreased by 5 U/L per year (*p* < 0.001); AST decreased by 3 U/L per year (*p* < 0.001)
29	Abad et al. [84]	ESG	RCT	16.5 months (72 weeks)	Liver biopsy, FibroScan	CAP showed a mean decrease in liver steatosis of 41.12 ± 49.41 dB/m (*p* = 0.067), which was observed in 81.3% of patients; NAS score decreased by 1.89 points (*p* = 0.544); steatosis decreased by 0.94 points (±0.87) (*p* = 0.033); MASH resolution happened in 27.8% (*p* = 0.36)	FibroScan (LSM) showed a mean decrease in liver stiffness of 5.63 ± 7.2 kPa (*p* = 0.017)	ALT decreased by 32.8 U/L at week 48 (*p* = 0.015) and by 27.7 U/L from baseline at week 72 (not significant); AST decreased by 23.6 U/L at week 48 and 21.3 U/L from baseline at week 72 (both not significant); GGT decreased by 47.1 U/L at week 48 (*p* = 0.015) and by 41.2 U/L from baseline at week 72 (*p* = 0.037)
30	Jagtap et al. [83]	ESG	Multicenter prospective cohort study-	12 months	NFS, HIS, FIB-4, APRI	HSI decreased by 9.9% at 6 months (*p* = 0.001) and by 12.1% from baseline at 12 months (*p* = 0.001)	FIB-4 decreased by 14.0% at 6 months (*p* = 0.174) and by 25.5% at 12 months compared to baseline (*p* = 0.013); APRI decreased by 20.1% at 6 months (*p* = 0.001) and by 34.1% at 12 months compared to baseline (*p* = 0.001); NFS decreased by 0.43 points at 6 months (*p* = 0.001) and by 0.78 points at 12 months compared to baseline (*p* = 0.001)	ALT levels decreased by 16.9% at 6 months (*p* = 0.006) and by 18.7% at 12 months compared to baseline (*p* = 0.022)
31	Nunes et al. [82]	ESG	Systematic review and meta-analysis	12 months	NFS, HIS	HSI decreased by 4.85 points (*p* < 0.00001)	NFS decreased by 0.50 points (*p* = 0.001)	ALT levels decreased by 16.5% (*p* = 0.0001)
